# Characterization of quinoa (*Chenopodium quinoa*) fermented by *Rhizopus oligosporus* and its bioactive properties

**DOI:** 10.1186/s13568-018-0675-3

**Published:** 2018-09-10

**Authors:** Jaewon Hur, Thi Thanh Hanh Nguyen, Namhyeon Park, Jeesoo Kim, Doman Kim

**Affiliations:** 1 Graduate School of International Agricultural Technology, Seoul National University, Pyeongchang, 25354 Republic of Korea; 2 Institutes of Food Industrialization, Institutes of Green Bio Science & Biotechnology, Seoul National University, Pyeongchang, 25354 Republic of Korea; 3 Center for Food and Bioconvergence, Seoul National University, Seoul, 08826 Republic of Korea

**Keywords:** Anti-inflammatory activity, Antioxidant activity, Fermentation, l-Carnitine, Quinoa, *Rhizopus oligosporus*

## Abstract

Quinoa is a pseudocereal that contains high quality protein, minerals, vitamins, polyphenols, and phytosterols. In this study, quinoa was fermented by *Rhizopus oligosporus* (*R. oligosporus*) up to 5 days and the functional compounds (l-carnitine, GABA, vanillic acid and gallic acid) were analyzed by LC/MS. The amounts of l-carnitine and GABA were 0.13 mg/kg and 540 mg/kg for nonfermented quinoa (NF), 3.15 mg/kg and 1040 mg/kg for fermented quinoa at 3 days (3F), and 1.54 mg/kg and 810 mg/kg for fermented quinoa at 5 days (5F). The vanillic acid and gallic acid were 1.3 and 0.1 mg/kg for NF, 1.55 and 2.37 mg/kg for 3F, and 1.83 and 0.84 mg/kg for 5F, respectively. Total phenolic contents and total flavonoids contents were 41 mg gallic acid (GAE)/kg and 13 mg quercetin equivalent (QE)/kg for NF, 74 mg GAE/kg and 16 mg QE/kg for 3F, and 80 mg GAE/kg and 19 mg QE/kg for 5F, respectively. Antioxidant activity (SC_50_) was 3.6 mg/mL for NF, 3.4 mg/mL for 3F, and 2.3 mg/mL for 5F. Nitric oxide production on RAW264.7 macrophages of fermented quinoa revealed 29% and 56% inhibition of nitric oxide production for NF and 5F, respectively. Therefore, fermented quinoa can be used as a healthy and valuable food product.

**Electronic supplementary material:**

The online version of this article (10.1186/s13568-018-0675-3) contains supplementary material, which is available to authorized users.

## Introduction

Quinoa (*Chenopodium quinoa*) has been widely cultivated in South America for 5000–7000 years (Vega-Gálvez et al. 2010). It has high nutritional properties and contents of antioxidant compounds; essential amino acids, vitamins, minerals, and unsaturated fatty acids (Carciochi et al. 2016). Lysine and methionine contents are high in quinoa, deficient in many legumes and rice, and also can be a precursor of l-carnitine synthesis (Koeth et al. 2013). l-carnitine is synthesized from lysine and methionine, serving an essential function in transportation of fatty acids into the mitochondrial compartment for β-oxidation and subsequent energy production (Bremer 1983). l-carnitine is abundant in red meat (5977 mg/kg beef) but in lesser amounts in crops (0.4 mg/kg wheat seed), and plants (2.9 mg/kg tomatoes, 0.8 mg/kg avocado) (Steiber et al. 2004). In addition, l-carnitine is an antioxidant compound that can prevent oxidative stress and regulates cellular respiration by nitric oxide (Brown 1999). As an antioxidant role of l-carnitine, mainly three enzymes (glutathione peroxidase, catalase, and superoxide dismutase) are prevented from peroxidative damage (Kalaiselvi and Panneerselvam 1998).

*Rhizopus oligosporus* (*R. oligosporus*) is a dominant fungus in special fermented soybean products like Indonesia teampeh. During the fermentation by *R. oligosporus*, the macromolecules were hydrolyzed by enzymes and coupled with the metabolism of the corresponding hydrolytic products to changes in the biochemical composition of food substrates (deReu et al. 1997; Handoyo and Morita 2006). Fermented soybean with *R. oligosporus* showed increase amounts of γ-aminobutyric acid (GABA), one of main fermented products from synthesizing from glutamate by glutamate decarboxylase (Dhakal et al. 2012; Handoyo and Morita 2006). In our previous study, *R. oligosporus* was used to produce l-carnitine and GABA in buckwheat by fermentation with no additional nutrients, and feed for chickens to gain high l-carnitine content in eggs (Park et al. 2016). The quinoa fermentation by using *R. oligosporus* have been reported (Matsuo 2005, 2006). However, these studies only focus on antioxidant activity of fermented quinoa. And there was no report for the improvement of l-carnitine and GABA in fermented quinoa as well as its biological and biochemical studies. In this regard, we fermented quinoa with *R. oligosporu*s DK1 to improve the quality of the food regarding the enhancement of l-carnitine, GABA, phenolic acid compounds, antioxidant and anti-inflammatory effects. White quinoa was selected for this study because it contains more antioxidative compounds than those of red and black quinoa (Masayo and Watanabe 2010).

## Materials and methods

### Microbial strain and culture condition

*Rhizopus microspores* var. *oligosporus* was obtained from our previous study (Park et al. 2016) and deposited as KCCM 11948P (Korean Culture Center of Microorganisms, Seoul, Korea). It was maintained on Potato Dextrose Agar (PDA, Difco, USA) plates.

### Preparation of fermented quinoa

*Rhizopus oligosporus* was cultured on PDA medium at 30 °C for 3 days to prepare spores (Park et al. 2016). White quinoa was purchased from KtFood (Seoul, Korea). 150 g quinoa was soaked in 150 mL water for 12 h and steamed for 20 min at 121 °C. Fermentation was conducted by inoculating 1 × 10^4^ spores/g steamed quinoa at 30 °C for 3–5 days. Fermented quinoa was lyophilized at − 10 to 0 °C under 20 Pa (Tokyo Rikakikai Co., Tokyo, Japan) for further study.

### Sample extraction

300 g lyophilized quinoa was extracted with 1 L of ethanol for 1 h at 28 °C in the shaking incubator and repeated seven times, then filtered using filter paper (8 micron, 11 cm) (Whatman LTD., Maidstone, England). Ethanol in the sample was removed by evaporation (Heidolph Instruments, Schwabach, Germany) with addition of 400 mL distilled water. The lipid layers from extracted sample were removed and the extracted sample were lyophilized for further study. Extraction yield was calculated as follows;

$$ {\text{Yield}}\, ( {{\text{g}}/100\, {\text{g}}}) \% = \frac{extract\,mass\,  ( {\rm g})}{{quinoa\,mass \, ( {300\, {\rm g}} )}} \times 100\,( \%) $$

### Scanning electron microscopy image analyses

Quinoa surfaces of sample prepared before and after fermentation were observed using a scanning electron microscope (SEM, TM 3030plus, Hitachi, Tokyo, Japan). Whole grain images were taken at magnification of ×100, 5.0 kV of accelerate voltage in secondary electron (SE) mode. At magnification ×1.0 k, fungi hypha was removed and observed at 15 kV of accelerate voltage in SE mode.

### Analyses of l-carnitine and GABA

LC/MS analysis was conducted by using the method as our previously described (Park et al. 2016). Samples were dissolved in water for analysis of l-carnitine or GABA. Samples were diluted with acetonitrile for l-carnitine and GABA, and all filtered using a 0.2 µm membrane (Sartorius AG, Gottingen, Germany). A 1 µL sample was injected into the LC/MS system (Waters, Milford, MA, USA); Waters Acquity H-Class system with Waters QDa detector, Waters Acquity UPLC BEH HILIC 1.7 µm, 2.1 mm × 100 mm column. Solvent A was 15 mM ammonium formate with 0.1% formic acid in distilled water and solvent B was 0.1% formic acid in acetonitrile. The temperature of column was maintained at 40 °C. The following elution gradient was applied for l-carnitine and GABA analyses; 0–3 min, 10% A; 3.1–5 min, 10–30% A; 5.1–6 min, 30–60% A; then a 4 min for equilibrium step. Electrospray ionization (ESI) was conducted with a positive with selective ion recording (SIR) (m/z 162 for l-carnitine and m/z 104 for GABA). Capillary energies were 1.5 kV. Cone voltage was 10 V for l-carnitine and 5 V for GABA. Acetonitrile (90%, v/v) was used as a blank. Calibration curves were prepared using the external standard method with l-carnitine concentrations ranged from 0.01 to 1 µg/mL, GABA ranged from 0.1 to 10 µg/mL. Linearity between concentrations of standards vs area was evaluated (*r*^2^ > 0.99).

### Analyses of phenolic acids

LC/MS analysis was conducted by using the method as previously described (Park et al. 2016). Samples were dissolved in DMSO for vanillic acid, chlorogenic acid, or gallic acid as 10 mg/mL. Samples were diluted with methanol for phenolic acids, and all filtered using a 0.2 µm membrane. A 1 µL sample was injected into the LC/MS system. Solvent A was distilled with water and solvent B was acetonitrile with 1 mL formic acid/L. The temperature of column was maintained at 40 °C. The following elution gradient was applied for phenolic acids analyses; 0–0.5 min, 95% A; 0.5–3 min, 95–70% A; 3–5 min, 70–0% A; 5–6 min, 0% A; then a 4 min for equilibrium step. ESI was conducted with a negative with SIR (m/z 169 for gallic acid, m/z 353 for chlorogenic acid, and m/z 167 for vanillic acid). Capillary energy was 0.8 kV for phenolic acids. Cone voltage was 10 V for phenolic acids and methanol was used as a blank. Calibration curves were prepared using the external standard method with phenolic acids concentration ranged from 0.1 to 10 µg/mL. Linearity between concentrations of standards vs area was evaluated (*r*^2^ > 0.99).

### Total phenolic contents analysis (TPC)

The total phenolic contents were determined by Folin ciocalteu’s method (Masayo and Watanabe 2010) with gallic acid as the standard (Sigma). 10 mg quinoa or fermented quinoa extracted powder was dissolved in 1 mL water. Each 120 µL of sample or gallic acid (0–50 µg/mL) was added into 96 wells plate and 15 µL of Folin ciocalteu’s reagent (Sigma) was mixed together for 3 min in dark condition. Then, 15 µL of 10% (w/v) Na_2_CO_3_ was added and reacted for 30 min in dark condition. The TPC were determined by spectrophotometry at 750 nm (SpectraMax M3, Molecular Devices, USA) and presented as gallic acid equivalent (GAE).

### Total flavonoid contents (TFC) analysis

TFC was determined by aluminum chloride colorimetric method (Chang et al. 2002) with quercetin as the standard (Sigma). 20 mg quinoa or fermented quinoa extracted powder was dissolved in 1 mL DMSO, and then fivefolds diluted in methanol to give final concentration as 1 mg/mL and volume of 2 mL. Each sample or quercetin (0–15 µg/mL) was mixed with 100 μL of 10% (w/v) AlCl_3_ and 100 μL of 0.1 mM CH_3_CO_2_K. TFC was determined by spectrophotometry at 415 nm using SpectraMax M3 and presented as quercetin equivalent (QE).

### Determination of DPPH radical scavenging activity

Antioxidant activities of quinoa or fermented quinoa were evaluated by 2,2-diphenyl-1-picrylhydrazyl (DPPH) radical scavenging method (Nguyen et al. 2015). Quinoa extracted powder were diluted in 70% ethanol and centrifuged at 13,572×*g* for 10 min. Supernatants were reacted with 100 μM DPPH (Sigma) in ethanol solution to give a final concentration of 0.2–7 mg quinoa extract/mL, then, kept at room temperature for 30 min in darkness. Absorbance of each sample was measured at 517 nm on a microplate reader, SpectraMax M3. DPPH radical-scavenging activity was converted into percentage of antioxidant activity using the following equation (Choi et al. 2018a):

$$ {\text{DPPH radcal}} - {\text{scavenging activity }}\left( \% \right) = \frac{{\left( {{\text{Absorbance of control}} - {\text{Absorbance of test sample}}} \right)}}{\text{Absorbance of control}} \times 100 $$

A linear regression curve was established to determine the amount of sample necessary to decrease 50% of the absorbance of DPPH (SC_50_ value). All analyses were conducted in duplicate. Results were expressed as mean ± standard error (SEM).

### Cell cytotoxicity tests

RAW264.7 mouse macrophage cell line was purchased from Korean Cell Line Bank (Seoul, Korea) and cultured in Dulbecco’s modified Eagle’s medium (DMEM, Gendepot, USA) supplemented with 10% (v/v) fetal bovine serum (FBS, Gendepot, USA), 100 U/mL penicillin and 100 µg/mL streptomycin (Invitrogen, USA) at 37 °C in 5% CO_2_ (Choi et al. 2018b; Maxwell et al. 2017). RAW264.7 macrophage cell was seeded on 96 wells plate at 2 × 10^4^ cells/well and cultured for 48 h. Cells were rinsed with phosphate-buffered saline (PBS) and then treated with quinoa or fermented quinoa extract in DMEM medium without Fetal bovine serum (FBS) ranging from 1.56 to 1600 µg/mL obtained by diluting quinoa or fermented quinoa extract with the culture medium. RAW 246.7 cells cultured in a medium without adding samples were used as controls. After 24 h at 37 °C, 90 µL of medium was mixed with 10 µL of Ez-CyTox solution (Daeil Lab Service, Seoul, Korea) and then incubated at 37 °C for 1 h. Absorbance was measured at 450 nm using SpectraMax M3. Percent viability was calculated as cell viability relative to the control.

### Measurement of nitric oxide production

Nitric oxide production was determined as previously described method (Kim et al. 1999). RAW 264.7 cells were seeded to 96 wells plate at 2 × 10^4^ cells/well and cultured at 37 °C for 48 h. The sample treated with 1 µg LPS/mL and 100 µM indomethacin was used as positive control. Cells were then treated with quinoa or fermented quinoa extract in DMSO ranging from 12.5 to 50 µg/mL without effects on cytotoxicity under testing, and cultured at 37 °C for 24 h. Then, 80 μL of culture supernatant was mixed with 80 µL of Griess reagent containing 1% (w/v) sulfanilamide in 5% (v/v) phosphoric acid, and 0.1% (w/v) naphthylethylenediamine, for 20 min, and absorbance was measured at 540 nm using SpectraMax M3. The amount of nitrite in the sample was evaluated from a standard curve generated with a sodium nitrite standard curve (0–500 µM in cell culture medium).

### Statistical analysis

Experimental results were statistically analyzed by t-test (IBM SPSS Statistics 22, IBM, USA). Values are presented as mean ± standard error of the mean (SEM). Significant differences between the groups were evaluated and indicated by different lower-case letters (p < 0.05 and p < 0.01).

## Results

### Scanning electron microscopic observation

**Fig. 1:**
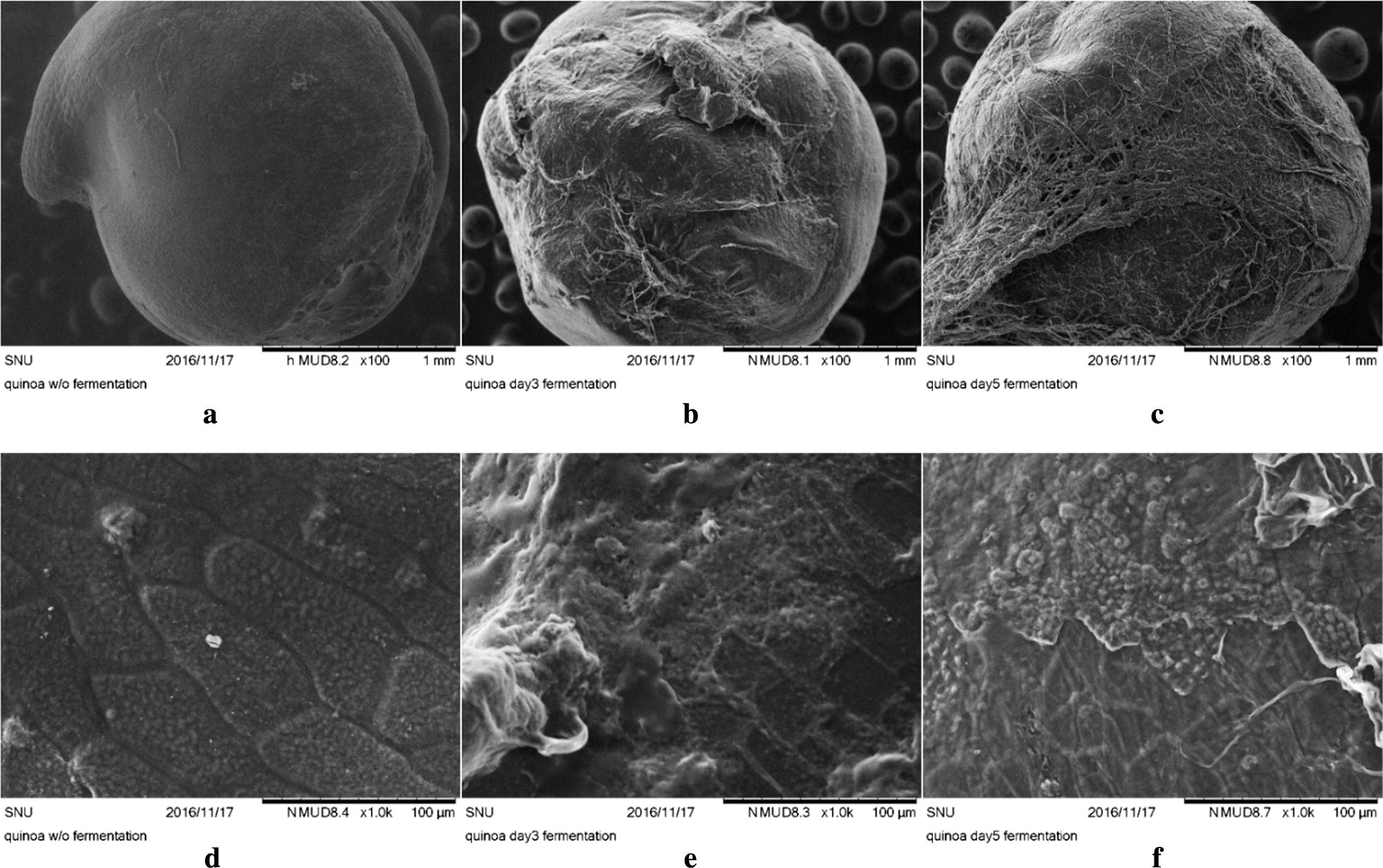
Scanning electron microscope of fermented quinoa. **a** NF, ×100; **b** 3F, ×100; **c** 5F, ×100; **d** NF, ×1000; **e** hypha removed 3F, ×1000; **f** hypha removed 5F, ×1000. (*NF* Non-fermented, 3F; 3 days, 5F; 5 days of fermented quinoa extracts)

The general quinoa and degradation of quinoa after fermentation were observed by the SEM. Figure 1 shows the surface of general and fermented quinoa. General quinoa (Fig. 1a) was observed with smooth surface. Fermented quinoa (Fig. 1b, c) was covered by *R. oligosporus* and mycelium was observed on fermented quinoa. The washed off fermented quinoa (Fig. 1e, f) revealed surface degradation comparable to the general quinoa (Fig. 1d), and exposed starch granule.

### Ethanol extraction of fermented quinoa

The extraction yields of NF, 3F, and 5F were 23.4%, 45.9%, and 39.1%, respectively. Among them, 3 days fermented quinoa showed highest extraction yield.

### Analyses of l-carnitine, GABA, and phenolic acids

The l-carnitine content was enhanced from 0.13 mg/kg to 3.15 and 1.54 mg/kg of quinoa extracts at 3F and 5F, respectively (Table 1). GABA was produced by the *R. oligosporus* fermentation, from 540 mg/kg to 1040 and 810 mg/kg of quinoa extracts at 3F and 5F, respectively (Table 1). Concentration of vanillic acids was increased during fermentation as 1.3, 1.55, and 1.83 mg/kg in NF, 3F, and 5F extracts, respectively (Table 1). Concentration of gallic acid was 0.01, 2.37, and 0.84 mg/kg in NF, 3F, and 5F extracts, respectively (Table 1). Chlorogenic acid was found 0.002 mg/kg for NF and 5F, but 0.03 mg/kg was detected at 3F extract (Table 1).

**Table 1:** l-carnitine, GABA and phenolic acids in regular and fermented quinoa

Group	NF	3F	5F
l-carnitine (mg/kg of extracts)	0.13	3.15 ± 0.06**	1.54 ± 0.06**
GABA (mg/kg of extracts)	540 ± 3	1040 ± 10**	810 ± 3**
Phenolic acids			
Vanillic acid (mg/kg of extracts)	1.3 ± 0.04	1.55 ± 0.06**	1.83 ± 0.06**
Gallic acid (mg/kg of extracts)	0.01	2.37 ± 0.08**	0.84 ± 0.02**
Chlorogenic acid (mg/kg of extracts)	0.002	0.03	0.002

(*NF* non-fermented, *3F* 3 days, *5F* 5 days of fermented quinoa extracts); (NF vs 3F and 5F, **p < 0.01)

### Total phenol content, total flavonoid content, and antioxidant activity of quinoa

Antioxidant activity was mainly investigated based on analysis of TPC, TFC, or DPPH radical-scavenging activity of each sample. After fermentation, TPC was increased from 41 mg GAE/kg to 74 and 80 mg GAE/kg of quinoa extracts at 3F and 5F, respectively (Table 2). TFC was increased from 13 mg QE/kg to 16 and 19 mg QE/kg of quinoa extract at 3F and 5F, respectively (Table 2). Antioxidant activity (SC_50_) of quinoa extracts prepared with NF, 3F, and 5F were 3.6, 3.4, and 2.3 mg/mL, respectively (Table 2, Additional file 1: Figure S1).

**Table 2:** Total phenolic and flavonoids contents and DPPH-radical scavenging activity of fermented quinoa extract

	NF	3F	5F
Total phenolic contents (mg GAE/kg of quinoa extract)	41 ± 1	74**	80 ± 1**
Total flavonoid contents (mg QE/kg of quinoa extract)	13	16**	19**
DPPH radical-scavenging activity (SC_50_ (mg/mL)	3.6	3.4 ± 0.5	2.3 ± 0.1**

(*NF* non-fermented, *3F* 3 days, *5F* 5 days of fermented quinoa extracts); (NF vs 3F and 5F, **p < 0.01)

### Cell viability of RAW264.7 cells

**Fig. 2:**
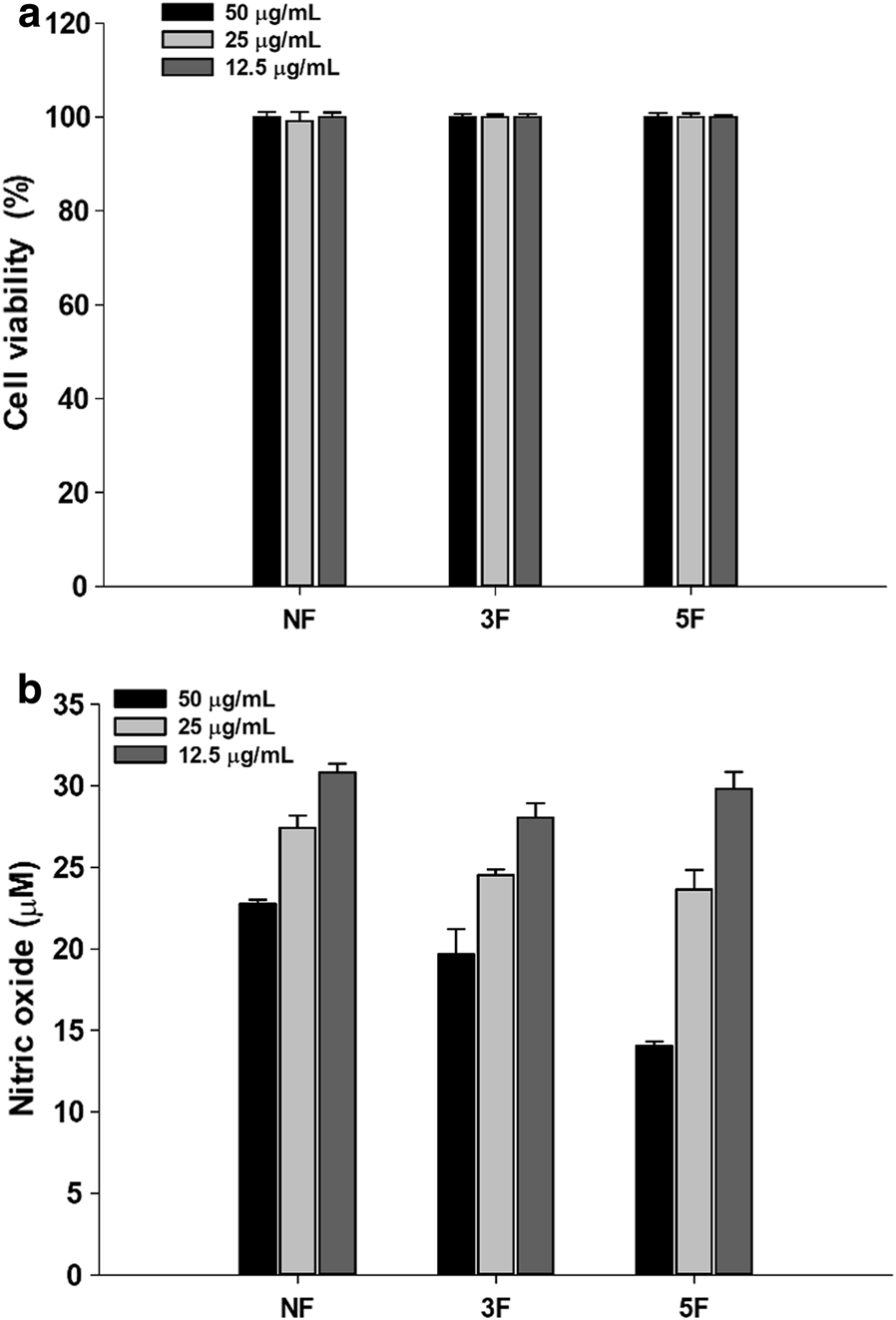
Cell viability (**a**) and nitric oxide production (**b**) on RAW264.7 of quinoa and fermented quinoa extract. (NF;Non-fermented, 3F; 3 days, 5F; 5 days of fermented quinoa extracts) (NF vs 3F and 5F, **p < 0.01)

Cell viabilities of RAW 264.7, macrophages cells, are shown in Fig. 2a. Cell viabilities were reached at 100% at the concentration of 100 µg/mL so that the nitric oxide assay was conducted from 50, 25, and 12.5 µg/mL (Fig. 2a).

### Nitric oxide production

Production of nitric oxide was investigated by lipopolysaccharides (LPS) stimulation. At the concentration of 50 μg/mL, the 5F extracts had significantly high anti-inflammatory activities. Production of nitric oxide was decreased 22.8, 19,7 and 14.0 µM in NF, 3F, and 5F extracts, respectively (Fig. 2b). These patterns were also shown at 25 μg/mL as 27.4, 24.5 and 23.6 µM of nitric oxide production in NF, 3F, and 5F extracts, respectively. As for 12.5 μg/mL, the nitric oxide was produced 30.8, 28.0 and 29.8 µM at NF, 3F, and 5F extracts, respectively. Since nitric oxide production is generated from inflammation, fermented quinoa had therapeutic abilities by reducing nitric oxide production.

## Discussion

Traditionally, quinoa has been cooked in salads, soups, porridges, and stews. Quinoa are found in forms of flakes, grains, and flours and have increasingly become incorporated into products such as noodles and energy bars. Recent developments of quinoa flour in small-scale products include bread, muffins, pasta, snacks, drinks, flakes, baby foods, beer and extrudates (Diaz et al. 2013; Matsuo 2006). In order to improve the functionalities, nutrition values and taste of quinoa, we fermented quinoa grains using *R. oligosporus.* Morphological characteristics by using the SEM reveals degradation of quinoa by *R. oligosporus* enzymes after fermentation (Fig. 1a–c). Raw quinoa seeds had polygonal granules (0.6–2.0 µm diameter) present singly and as spherical aggregates (Ruales and Nair 1993). After fermentation, the polygonal granule was hardly observed and degraded into low-molecule-substances that brought different biological values to fermented quinoa (Fig. 1b, c). Degradation of cell structures of fermented quinoa was due to lipases, amylases, protease, and glucoamylase that produced from *R. oligosporus* (Handoyo and Morita 2006; Jin et al. 1999). The 3F and 5F quinoa extracts had higher content of l-carnitine (24.1 and 11.8 times) than non-fermented quinoa. Handoyo and Morita (2006) reported that *R. oligosporus* hydrolyzed protein into amino acids and small peptides. In other study, Matsuo (2006) reported that the amount of lysine was increased from non-detection to 0.31 g/kg dry quinoa and lysine from 0.11 to 0.51 g/kg dry quinoa during *R. oligosporus* fermentation. l-carnitine is synthesized from lysine and methionine thus the synthesis of l-carnitine depends on the amount of lysine and methionine in quinoa. The GABA contents in fermented quinoa showed 1.9 and 1.5 times higher than that of NF. GABA is synthesized from glutamate catalyzing by glutamate decarboxylase while quinoa contained high glutamate (0.71 g/kg dry quinoa) (Matsuo 2006). Fermented quinoa extract revealed 36.1% enhanced anti-oxidative activity in DPPH-radical scavenging activity level. This result was agreed with previous reported by Mastuo (2006). The enhancement of antioxidant activities probably resulted in the increased amounts of phenolic compounds by the fermentation. Contents of phenolic acids such as vanillic acids, gallic acids, chlorogenic acids, and TPC, TFC were also changed and possibly effected to improvement of DPPH-radical scavenging activity. Vanillic acid was known for main phenolic acids in quinoa, and other phenolic acids were also analyzed such as gallic acids and chlorogenic acids. Vanillic acids contents were increased by fermentation as 1.3–1.83 mg/kg by NF and 5F, respectively. In case of gallic acids were increased at 3F by 2.37 mg/kg from 0.01 mg/kg of NF, but decreased at 5F by 0.84 mg/kg compared to 3F. As for chlorogenic acid, the pattern was similarly revealed as gallic acids for increased amount at 3F by 0.03 mg/kg from 0.02 mg/kg of NF, and decreased at 5F as 0.02 mg/kg from 3F. It is probable that phenolic acids are derivatives for other phenolic acids (Rice-Evans et al. 1996). In this study, TPC (Table 2) were enhanced (1.8 and 2.0 times increase at 3F and 5F) during the *R. oligosporus* fermentation and were probably obtained from formation of higher contents of phenolic compounds such as vanillic acids, gallic acids and chlorogenic acids. McCue and Shetty (2003) reported that α-amylase and endogenous carbohydrate-cleaving enzymes activities had a role to generate polyphenols from carbohydrates-conjugated phenolic compounds. *R. oligosporus* is a known strain to produce β-glucosidase, β-glucuronidase and xylanase when degrade the cell wall matrix (Huynh et al. 2014). Thus, it was probably metabolized with the bioconversion of phenolic compounds by the fermentation leads the cell-wall degrading enzymes to hydrolysis of glycosidic bonds and produces bound phenolics and aglycone forms (Huynh et al. 2014). Also, TFC (Table 2) were enhanced by 1.2 and 1.5 times increase at 3F and 5F. The fermentation processes releasing phenolic compounds from plant matrixes followed by the metabolic pathways of flavonoids: glycosylation, deglycosylation, ring cleavage, methylation, glucuronidation, and sulfate conjunction which are way of producing new bioactive compounds (Huynh et al. 2014). These increases in phenolic compounds effected to the enhancement of antioxidative activity (Kaur and Kapoor 2002). Increase of flavonoids contents has also influenced on DPPH-radical scavenging activity resulting in higher antioxidant activity of fermented quinoa extracts than non-fermented ones. Since antioxidant properties of fermented quinoa extracts were found, anti-inflammatory effect on mammalian cells was further investigated. Anti-inflammatory function of fermented quinoa extract was studied on RAW 264.7, macrophages, with LPS-stimulation. Nitric oxide production was inhibited 29.3% for NF, 38.9 for 3F and 56.4% for 5F, resulting in improvement of 192.6% in anti-inflammatory activity (Fig. 2b). Kim et al. (1999) reported that the flavonoids inhibit NO production in lipopolysaccharide-activated RAW264.7 cells and reduce of iNOS enzyme expression. Thus, the increase anti-inflammatory effect of fermented quinoa compared to the regular quinoa due to increasing of total phenol and total flavonoids contents in fermented quinoa by *R. oligosporus*. Biscuits made by substituting 20% of the flour with *R. oligosporus* fermented quinoa powder increased its iron and α-tocopherol contents by a factor more than 2.5 and scored high in sensory analysis of flavor hardness and taste in soft biscuits (Matsuo 2006). The absorption of iron from *R. oligosporus* fermented quinoa was higher than that of quinoa powder in rats for partial digestion of phosphoric compounds (Matsuo 2006). Therefore, the increased concentration of l-carnitine and GABA in fermented quinoa by *R. oligosporus* could be improved by the beneficial functionalities and nutritional values in fermented quinoa supplemented food materials.

In conclusion, fermented quinoa extract has effective antioxidant and anti-inflammatory activities. These activities may be due to presence of phenolic compounds, flavonoids and the other products produced during fermentation by *R. oligosporus*. Although the 3rd day fermentation revealed optimal conditions to produce l-carnitine and GABA, the 5-day fermented quinoa extract had higher TPC, TFC, antioxidant activity and improved reduction in inflammation than regular quinoa extract. In this regard, fermented quinoa can be used as a healthy and valuable food product.

## Additional file

**Additional file 1: Fig. S1.** DPPH radical scavenging of nonfermented quinoa (NF), 3-day fermented quinoa (3F), 5-day fermented quinoa (5F).

## Abbreviations

*R. oligosporus*: *Rhizopus oligosporus*
GABA: γ-aminobutyric acid
NF: nonfermented quinoa
3F: fermented quinoa at 3 days
5F: fermented quinoa at 5 days
GAE: gallic acid equivalent
QE: quercetin equivalent
PDA: potato dextrose agar
SEM: scanning electron microscope
DMSO: dimethyl sulfoxide
LC/MS: liquid chromatography–mass spectrometry
TPC: total phenolic content
TFC: total flavonoid content
DPPH: 2,2-diphenyl-1-picryhydrazyl
DMEM: Dulbecco’s modified Eagle’s medium
