# Supplementary material for: Characterization of quinoa (Chenopodium quinoa) fermented by Rhizopus oligosporus and its bioactive properties
Source: AMB Express. 2018 Sep 10;8:143. doi: 10.1186/s13568-018-0675-3 (PMC6134574; doi:10.1186/s13568-018-0675-3)
Supplement: Supplementary file 1 — Additional file 1: Fig. S1. DPPH radical scavenging of nonfermented quinoa (NF), 3-day fermented quinoa (3F), 5-day fermented quinoa (5F). [file 13568_2018_675_MOESM1_ESM.docx]

**AMB Express**

**Additional materials**

**Characterization of quinoa (*Chenopodium quinoa*) fermented by *Rhizopus oligosporus* and its bioactive properties**

Jaewon Hur^1^, Nguyen Thi Thanh Hanh^2^, Namhyeon Park^2^, Jeesoo Kim^1^, Doman Kim^1,2^

^1^ Graduate School of International Agricultural Technology, Seoul National University, Pyeongchang, 25354, Republic of Korea. Email: [willbe17@snu.ac.kr](mailto:willbe17@snu.ac.kr) (JW Hur); [j_k084@snu.ac.kr](mailto:j_k084@snu.ac.kr) (JS Kim); [kimdm@snu.ac.kr](mailto:kimdm@snu.ac.kr) (D Kim)

^2^ Institutes of Food Industrialization, Institutes of Green Bio Science & Biotechnology, Seoul National University, Pyeongchang, 25354, Republic of Korea. Email: [hara2910@snu.ac.kr](mailto:hara2910@snu.ac.kr) (TTH Nguyen); [sayparknh@naver.com](mailto:sayparknh@naver.com) (NH Park).

^*^ Corresponding author.

E-mail: kimdm@snu.ac.kr Tel: +82-33-339-5720; Fax: +82-33-339-5716


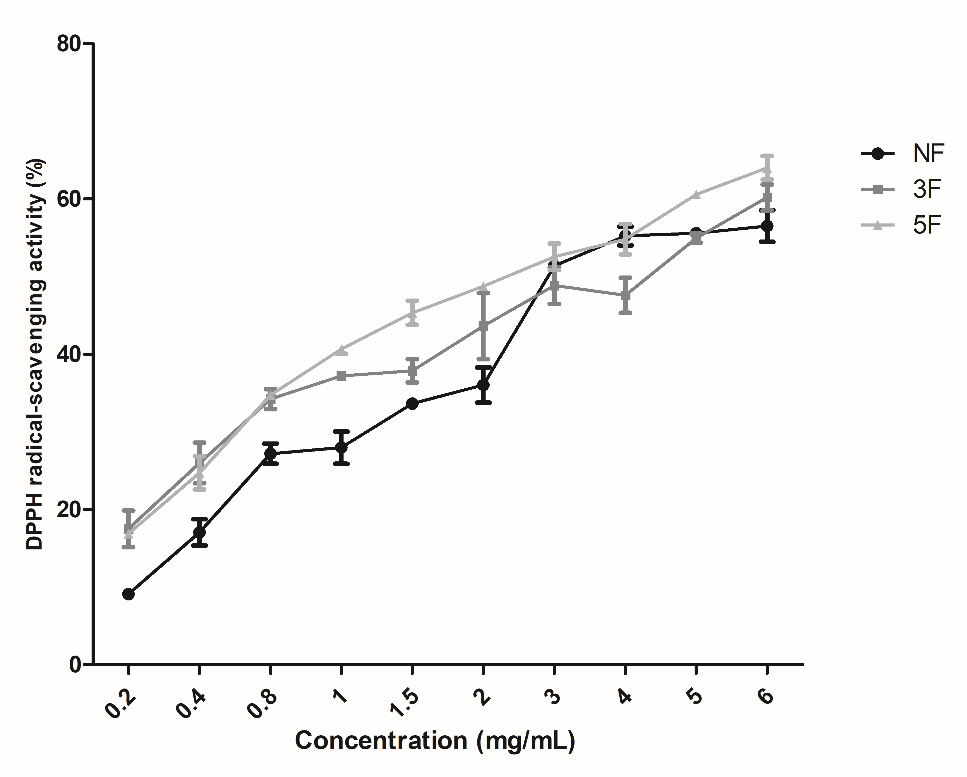


**Figure S1.** DPPH radical scavenging of nonfermented quinoa (NF), 3-day fermented quinoa (3F), 5-day fermented quinoa (5F).
